# Variation in *KRTAP6-1* affects wool fibre diameter in New Zealand Romney ewes

**DOI:** 10.5194/aab-62-509-2019

**Published:** 2019-08-13

**Authors:** Wenhao Li, Hua Gong, Huitong Zhou, Jiqing Wang, Shaobin Li, Xiu Liu, Yuzhu Luo, Jon G. H. Hickford

**Affiliations:** 1 Gansu Key Laboratory of Herbivorous Animal Biotechnology, Faculty of Animal Science and Technology, Gansu Agricultural University, Lanzhou 730070, China; 2 International Wool Research Institute, Gansu Agricultural University, Lanzhou 730070, China; 3 Gene-marker Laboratory, Faculty of Agricultural and Life Sciences, Lincoln University, Lincoln 7647, New Zealand

## Abstract

Variation in *KRTAP6-1* has been reported to affect wool fibre traits in Merino cross-breed sheep and Chinese Tan sheep, but little is known about whether these effects persist in other breeds. In this study, variation in *KRTAP6-1* was investigated in 290 New Zealand (NZ) Romney ewes sired by 16 different rams. Polymerase chain reaction single-stranded conformational polymorphism (PCR-SSCP) analysis revealed four variants (A, B, E and F) of *KRTAP6-1*, and nine genotypes (*AA*, *AB*, *AE*, *AF*, *BB*, *BE*, *BF*, *EE* and *FF*) in these ewes. Among the 243 ewes that had genotypes with a frequency of over 5 % (i.e. *AA*, *AB* and *BB*), the presence of A was found to be associated with reduced mean fibre diameter (MFD) and increased coefficient of variation in fibre diameter (CVFD), whereas the presence of B had a trend of association with decreased coarse edge measurement (CEM). A genotype effect was also detected for MFD and CVFD. No associations were detected for fibre diameter standard deviation (FDSD), mean fibre curvature (MFC) and medulation. These results suggest that variation in *KRTAP6-1* affects wool fibre diameter in NZ Romney ewes, confirming the finding in Merino cross-breed sheep.

## 1 Introduction

Keratin-associated proteins and keratin intermediate filament proteins are the major structural proteins of wool, and they cross-link to form the skeleton of wool fibres (Powell and Rogers, 1997). The keratin-associated proteins (KAPs) are classified into three groups according to their amino acid composition: the high sulfur family (HS; ≤30 mol % cysteine), the ultra-high sulfur family (UHS; >30 mol % cysteine) and the high glycine and tyrosine family (HGT; 35–60 mol % glycine and tyrosine) (Gong et al., 2016). Of these, the HGT-KAPs are the first group of KAPs to be produced in active wool follicles, and soon after the synthesis of the keratins (Rogers, 2006). They vary considerably in abundance between and within sheep breeds (Gillespie, 1990).

The KAP6 family is a diverse family from the HGT-KAP group. The family has five gene members (*KRTAP6-1, KRTAP6-2, KRTAP6-3, KRTAP6-4* and *KRTAP6-5*) in sheep (Zhou et al., 2016), and they are located on chromosome 1. They are clustered with six other HGT-KAP genes (*KRTAP7-1*, *KRTAP8-1*, *KRTAP8-2*, *KRTAP20-1*, *KRTAP20-2* and *KRTAP22-1*) (Bai et al., 2018; Gong et al., 2014, 2019; Li et al., 2017a) and six HS-KAP genes (*KRTAP11-1*, *KRTAP13-1*, *KRTAP15-1*, *KRTAP24-1*, *KRTAP26-1 *and *KRTAP28-1*) (Bai et al., 2019; Gong et al., 2011a, 2012b; Wang et al., 2017; Zhou et al., 2012). Five variant sequences have been identified at the *KRTAP6-1* locus and this variation has been reported to be associated with various mean fibre diameter (MFD)-associated traits in Merino cross-breed sheep (Zhou et al., 2015), and fibre length and crimping in early life in Chinese Tan sheep (Tao et al., 2017). These findings suggest variation in *KRTAP6-1* affects wool traits, but little is known about whether the effect is preserved across other breeds, including breeds that produce strong (or high MFD) wool.

The New Zealand (NZ) Romney sheep is a dual-purpose breed for meat and wool production. It is the most common sheep breed in NZ and accounts for over 47 % of the 27.4 million sheep wintered-over in June 2017 (Beef and Lamb New Zealand, 2018). The NZ Romney wool typically has a MFD of over 36 µm.

In this study, variation in *KRTAP6-1* was investigated in NZ Romney ewes using Polymerase chain reaction single-stranded conformational polymorphism (PCR-SSCP) analysis, and its association with six different wool traits was analysed to determine whether the *KRTAP6-1* variation affected these traits in this strong-wool breed.

## 2 Materials and methods

This study was carried out in accordance with the Animal Welfare Act 1999 (NZ Government), and the collection of sheep blood drops by nicking sheep ears is covered by Sect. 7.5, Animal Identification, of the Sheep and Beef Cattle Code of Welfare 2010 (NZ Government).

### 2.1 Sheep blood and wool sample collection and preparation

Two hundred and ninety NZ Romney ewes from 16 different sires sourced from seven farms in New Zealand were investigated initially. A blood sample from each ewe was collected onto TFN paper (Munktell Filter AB, Sweden) and genomic DNA was purified from dried blood spots using a two-step purification method described in Zhou et al. (2006).

A wool sample from the mid-side region of each ewe was collected at “hogget shearing”, with ages ranging from 15 to 17 months old, depending on the farm. These samples were measured for mean fibre diameter (MFD), fibre diameter standard deviation (FDSD), coefficient of variation in fibre diameter (CVFD), coarse edge measurement (CEM; the percentage of fibres that are 10 µm greater than the mean fibre diameter), mean fibre curvature (MFC), and medulation. The measurements of these wool traits were undertaken by Pastoral Measurements Limited (Timaru, NZ).

### 2.2 Genotyping of ovine *KRTAP6-1*

A fragment of *KRTAP6-1* containing the entire coding region was amplified using the PCR primers described in Zhou et al. (2015). These primers were 5${}^{\prime }$-TCTACCCGAGAACAACCTC-3${}^{\prime }$ (forward) and 5${}^{\prime }$-AGGCAAGTCTTTAGTAGGAC-3${}^{\prime }$ (reverse), and they were synthesized by Integrated DNA Technologies (Coralville, IA, USA). PCR amplification was performed using a 15 µL reaction consisting of a 1.2 mm punch of genomic DNA from the TFN card, 0.25 µM of each primer, 150 µM of each deoxynucleoside triphosphate (dNTP) (Bioline, London, UK), 2.5 mM ${\mathrm{Mg}}^{2+}$, 0.5 U of *Taq* DNA polymerase (Qiagen, Hilden, Germany) and 1× the reaction buffer supplied with the enzyme. The thermal profile included an initial denaturation at 94 ${}^{\circ }$C for 2 min, followed by 35 cycles of 94 ${}^{\circ }$C for 30 s, 59 ${}^{\circ }$C for 30 s and 72 ${}^{\circ }$C for 30 s, and a final extension step at 72 ${}^{\circ }$C for 5 min.

The PCR amplicons were subject to single-stranded conformational polymorphism (SSCP) analysis to differentiate variants. A 1 µL aliquot of each PCR amplicon was mixed with 7 µL of loading dye (98 % formamide, 10 mM EDTA, 0.025 % bromophenol blue, 0.025 % xylene cyanol) and denatured at 90 ${}^{\circ }$C for 5 min. The sample was rapidly cooled on wet ice and then loaded onto a 16cm×18 cm, 12 % polyacrylamide (acrylamide–bisacrylamide 37.5:1) gel containing 1 % glycerol. Electrophoresis was carried out in 0.5× TBE buffer at 330 V and 12 ${}^{\circ }$C for 16 h, and the gel was silver stained using the method by Byun et al. (2009).

### 2.3 Sequencing of variants and sequence analysis

Polymerase chain reaction amplicons representing different SSCP banding patterns from sheep that appeared to be homozygous were sequenced in both directions at the Lincoln University DNA sequencing facility (Lincoln University, New Zealand). Variants that were only found in heterozygous ewes were sequenced using an approach described by Gong et al. (2011b). Briefly, a band corresponding to the variant was removed as a slice from the polyacrylamide gel, mashed-up and then used as a template for re-amplification with the original primers. This second amplicon was then sequenced directly.

**Figure 1:**
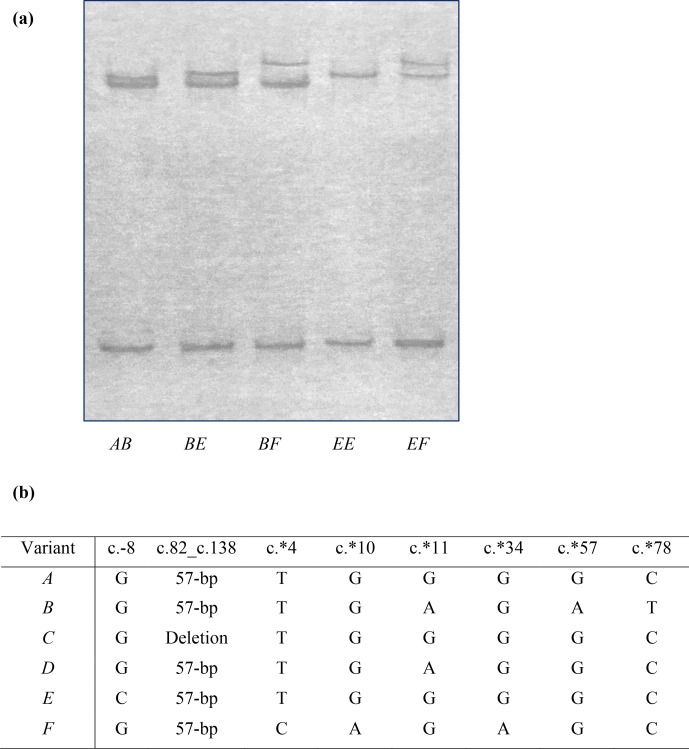
Polymorphism of ovine *KRTAP6-1*. **(a)** Four variants (A, B, E and F) of ovine *KRTAP6-1* were detected in New Zealand Romney ewes by PCR single-stranded conformational polymorphism analysis. **(b)** Sequence comparison of the four ovine *KRTAP6-1* variants identified in this study, and those previously identified variants (C and D), revealed three new single-nucleotide polymorphisms (SNPs) at c.${}^{*}$4, c.${}^{*}$10 and c.${}^{*}$34.

DNA sequence alignments, and translation to putative amino acid sequences (this assumes the gene is expressed) were carried out using DNAMAN (version 5.2.10, Lynnon BioSoft, Vaudreuil, Quebec, Canada).

### 2.4 Statistical analyses

Statistical analysis was performed using Minitab version 16 (Minitab Inc., State College, PA, USA). Association analyses were only carried out for the ewes that had variants and genotypes with frequencies of over 5 % (and therefore with sufficient sample size) and the ewes with other genotypes were removed from the association study.

General linear mixed models (GLMMs) were used to assess the effect of the absence or presence of individual variants on the wool traits, and also compare various wool traits between ewes with different genotypes. Bonferroni corrections were applied to these genotype models to reduce the chance of obtaining false positive results during the multiple comparisons. Sire was incorporated as random factors in the models. As each farm had different sires, the ewes from different farms were therefore from different sires; accordingly, the wool samples from different sires were taken at different ages. This meant that sire, farm and ewe age were confounded. Sire had the greatest effect on the wool traits, and therefore only sire was included in the models. All wool samples were collected from ewes, hence gender was not fitted into the models.

## 3 Results

### 3.1 Polymorphism of *KRTAP6-1* in NZ Romney ewes

Four unique PCR-SSCP patterns were detected among the NZ Romney ewes (Fig. 1a). Sequencing of amplicons representative of these banding patterns revealed three previously identified *KRTAP6-1* variants A, B and E (Zhou et al., 2015; Tao et al., 2017) and one newly identified variant named variant F. The detection of variant F resulted in the definition of three new single-nucleotide polymorphisms (SNPs) at c.${}^{*}$4, c.${}^{*}$10 and c.${}^{*}$34, and these SNPs were in linkage (Fig. 1b). Two previously identified variants, C and D (Zhou et al., 2015), were not found in these NZ Romney ewes.

Nine genotypes were observed, with the genotypes and frequencies being *AA* (17.5 %), *AB* (43.9 %), *AE* (2.6 %), *AF* (3.4 %), *BB* (22.5%), *BE* (3.9 %), *BF* (4.7 %), *EE* (0.5 %) and *FF* (1.0 %). These gave varying frequencies of 42.4 %, 48.7 %, 3.8 % and 5.1 % for A, B, E and F, respectively.

**Table 1:** Association between the presence or absence of ovine *KRTAP6-1* variants and wool traits in New Zealand Romney ewes.

Trait${}^{1}$	Variant${}^{2}$	Mean ± SE${}^{3}$		P value
		Presence	Absence	
MFD (µm)	A	35.6±0.20	36.7±0.30	0.002
	B	35.9±0.19	36.0±0.37	0.678
FDSD (µm)	A	8.6±0.08	8.5±0.13	0.319
	B	8.5±0.08	8.8±0.14	0.166
CVFD (%)	A	24.2±0.22	23.1±0.34	0.005
	B	23.8±0.21	24.3±0.41	0.204
CEM (%)	A	11.8±0.24	11.1±0.36	0.120
	B	11.4±0.22	12.2±0.43	0.099
MFC (${}^{\circ }$ mm${}^{-1}$)	A	62.6±0.45	62.2±0.70	0.603
	B	62.2±0.43	63.5±0.82	0.169
Medulation (%)	A	1.4±0.17	1.0±0.26	0.140
	B	1.4±0.16	1.1±0.31	0.386

${}^{1}$ MFD – mean fibre diameter; FDSD – fibre diameter standard deviation; CVFD – coefficient of variation in fibre diameter; CEM – coarse edge measurement (the percentage of fibres that are 10 µm greater than the mean fibre diameter); MFC – mean fibre curvature. ${}^{2}$ Of 243 Romney ewes that had common genotypes (i.e. *AA*, *AB* or *BB*), A was present in 179 ewes and absent in 64 ewes, and B was present in 192 ewes and absent in 51 ewes. ${}^{3}$ Estimated marginal means and standard errors (SE) of those means from general linear mixed models, with P<0.05 being shown in bold.

**Table 2:** Association of ovine *KRTAP6-1* genotypes and wool traits in New Zealand Romney ewes.

Trait${}^{1}$	Mean ± SE${}^{2}$			P value
	*AA* (n=51)	*AB* (n=128)	*BB* (n=64)	
MFD (µm)	$36.0\pm 0.36^{\text{ab}}$	$35.4\pm 0.22^{\text{b}}$	$36.7\pm 0.30^{\text{a}}$	0.003
FDSD (µm)	8.8±0.15	8.6±0.09	8.5±0.13	0.307
CVFD (%)	$24.4\pm 0.40^{\text{a}}$	$24.1\pm 0.25^{\text{ab}}$	$23.1\pm 0.34^{\text{b}}$	0.016
CEM (%)	12.2±0.43	11.6±0.27	11.1±0.36	0.128
MFC (${}^{\circ }$ mm${}^{-1}$)	63.5±0.83	62.3±0.51	62.1±0.70	0.383
Medulation (%)	1.1±0.31	1.6±0.19	1.0±0.26	0.147

${}^{1}$ MFD – mean fibre diameter; FDSD – fibre diameter standard deviation; CVFD – coefficient of variation in fibre diameter; CEM – coarse edge measurement (the percentage of fibres that are 10 µm greater than the mean fibre diameter); MFC – mean fibre curvature. ${}^{2}$ Estimated marginal means and standard errors (SE) of those means from general linear mixed models. Means within rows that do not share a superscript letter are different at P<0.05, and are shown in bold.

### 3.2 Association between *KRTAP6-1* variation and wool traits

Of the 243 ewes that had the common genotypes *AA*, *AB* and *BB* (occurring at frequencies of over 5 %), the presence of variant A was found to be associated with a decrease in MFD and an increase in CVFD and the presence of B was found to have a trend of association with decreased CEM (Table 1). Wool from the *AA* ewes had a higher CVFD than *BB* ewes, with *AB* ewes being intermediate (Table 2). Wool produced by *BB* ewes had higher MFD than *AB* ewes but was not different to *AA* ewes (Table 2). No associations were detected for FDSD, MFC or medulation.

## 4 Discussion

This study describes genetic variation in ovine *KRTAP6-1* in 15–17-month-old NZ Romney ewes and its association with MFD-associated wool traits in this strong-wool breed. At this age, wool measurement is important, as in most NZ Romney sheep production systems; this is when ewe selection decisions are commonly made and the ewes kept after selection are mated for the first time, entering the main ewe flock.

The detection of three previously identified and one newly identified variant, in these NZ Romney ewes, suggests *KRTAP6-1* exhibits a moderate level of variation in this breed but the nature of the variation in this breed appears to be different to that reported in Merino cross-breed sheep (Zhou et al., 2015) and Chinese Tan sheep (Tao et al., 2017). In particular, the C variant that contains a 57 bp deletion, and that was found to be associated with higher MFD, FDSD and CVFD in Merino cross-breed sheep (Zhou et al., 2015) was not found in these NZ Romney ewes. Variant D that occurred at a frequency of over 10 % in Chinese Tan sheep, and was found to be associated with increased straightened fibre length at birth (Tao et al., 2017), was also not found in the NZ Romney ewes. Instead a new variant (F) was detected.

The detection of a new variant brings the number of ovine *KRTAP6-1* variants from five to six, and the number of SNPs from four to seven in a 343 bp fragment of the gene (excluding the primer binding regions). This gives a density of over 20 SNPs per kilobase pair, which is much higher than the average density of 4.9 SNPs per kilobase pair across the sheep genome (Kijas et al., 2009). What is more, it might be that more variants are identified when more sheep from more breeds are investigated. These SNPs, together with the presence of 57 bp indel (Zhou et al., 2015) suggest that ovine *KRTAP6-1* is a polymorphic gene.

Sequence variation has been reported in many other ovine *KRTAP* genes (Gong et al., 2012a, 2019; Li et al., 2017a, b, 2018), but the sequence variation identified in *KRTAP6-1* has some unique features. First is the location of the sequence variation. All of the SNPs identified in ovine *KRTAP6-1* are located either upstream or downstream of the coding region, but not in the coding region itself. The absence of coding region SNPs has not been reported for any of the other ovine *KRTAP* genes identified (Gong et al., 2016, 2019; Li et al., 2017a, b, 2018), except *KRTAP8-2*, which has only one SNP at a position 21 bp upstream of the TATA box (Gong et al., 2014). The unusual location of SNPs suggests that ovine *KRTAP6-1* may be constrained as regards variation in its protein structure and/or function, but possibly exhibit flexibility in its level of expression.

The second notable feature is the linkage of the SNPs. For example, linkage is observed for the variation at c.${}^{*}$4, c.${}^{*}$10 and c.${}^{*}$34 and further linkage is detected for the variations at c.${}^{*}$57 and c.${}^{*}$78. The linkage could suggest that gene conversion may have occurred at a location downstream of the coding region and that this may have led to the generation of these SNPs. Gene conversion has been described for HS-*KRTAP1-n* (Rogers et al., 1994; Zhou et al., 2019); for the *KRTAP1-n* gene, conversion is observed within the coding region and appears to be the mechanism responsible for the concerted evolution of the *KRTAP1-n* coding region (Zhou et al., 2019).

The detection of variant and genotype associations with MFD and CVFD, but not with FDSD, suggests that variation in *KRTAP6-1* affects the average of the fibre diameters, but not the distribution of individual fibre diameters around that average. This is because CVFD=FDSD/MFD×100 %, and hence a decrease in MFD would lead to an increase in CVFD, if FDSD is unchanged. The failure to detect a difference in MFD for genotype *AA* compared to *AB* and *BB*, a situation that seems rather odd, may be due to a number of factors. Firstly, it may be an effect of sample size, as genotype *AA* was only found in 51 ewes, which is the smallest group among the genotypes used for the association analyses. Second, may be the level of variation observed for the wool traits. Genotype *AA* ewes had higher standard errors (SEs) for the adjusted means for all the wool traits compared to *AB* or *BB* ewes. High SEs may suggest higher levels of variation in traits, and suggesting that some uncontrolled variable is unaccounted for. For example, variant A, as described in this breed, might actually represent more than one form of the gene, with the difference between those forms occurring outside of the amplified region. Given that variation in *KRTAP6-1* described here is located outside of the coding region, it remains possible that even more variation of consequence to the activity of the gene occurs in regions further upstream or downstream. It is also possible that variant A may be linked to variation in other proximal *KRTAP* genes, and consequently there is some kind of haplotype effect. This would once again require further investigation.

The effect of the *KRTAP6-1* variation detected in the NZ Romney ewes appears to be different to that detected in Merino cross-breed sheep, in which FDSD and prickle factor (comparable to CEM described for coarse wool) are also affected (Zhou et al., 2015). This suggests that the role that *KRTAP6-1* plays in regulating wool fibre traits may differ in fine- and strong-wool breeds. Zhang et al. (2017) reported that *KRTAP6-1* was differentially expressed in Chinese Merino (fine wool) and Small Tailed Han (strong wool) sheep, with a higher level of expression in the Merino sheep compared to the Small Tailed Han sheep. Reduced expression of *KRTAP6-1* may lead to a reduced effect on wool traits, which appears to be consistent with the results obtained in NZ Romney ewes. This would require further investigation; nevertheless, the results from this study demonstrate that further sequence variation occurs in ovine *KRTAP6-1*, and that this sequence variation also affects wool fibre diameter traits.

## Data Availability

The original data are available upon request to the corresponding authors.
